# Autophagy-Based Hypothesis on the Role of Brain Catecholamine Response During Stress

**DOI:** 10.3389/fpsyt.2020.569248

**Published:** 2020-09-17

**Authors:** Fiona Limanaqi, Carla Letizia Busceti, Francesca Biagioni, Francesco Fornai, Stefano Puglisi-Allegra

**Affiliations:** ^1^ Department of Translational Research and New Technologies on Medicine and Surgery, University of Pisa, Pisa, Italy; ^2^ IRCCS Neuromed, Pozzilli, Italy

**Keywords:** locus coeruleus, ventral tegmental area, dopamine, norepinephrine, drug addiction, sensitization, corticotrophin-releasing factor, brain-derived neurotrophic factor

## Abstract

Stressful events, similar to abused drugs, significantly affect the homeostatic balance of the catecholamine brain systems while activating compensation mechanisms to restore balance. In detail, norepinephrine (NE)- and dopamine (DA)-containing neurons within the locus coeruleus (LC) and ventral tegmental area (VTA), are readily and similarly activated by psychostimulants and stressful events involving neural processes related to perception, reward, cognitive evaluation, appraisal, and stress-dependent hormonal factors. Brain catecholamine response to stress results in time-dependent regulatory processes involving mesocorticolimbic circuits and networks, where LC-NE neurons respond more readily than VTA-DA neurons. LC-NE projections are dominant in controlling the forebrain DA-targeted areas, such as the nucleus accumbens (NAc) and medial pre-frontal cortex (mPFC). Heavy and persistent coping demand could lead to sustained LC-NE and VTA-DA neuronal activity, that, when persisting chronically, is supposed to alter LC-VTA synaptic connections. Increasing evidence has been provided indicating a role of autophagy in modulating DA neurotransmission and synaptic plasticity. This alters behavior, and emotional/cognitive experience in response to drug abuse and occasionally, to psychological stress. Thus, relevant information to address the role of stress and autophagy can be drawn from psychostimulants research. In the present mini-review we discuss the role of autophagy in brain catecholamine response to stress and its dysregulation. The findings here discussed suggest a crucial role of regulated autophagy in the response and adaptation of LC-NE and VTA-DA systems to stress.

## Introduction

Stress is one consequence of challenges to the organism produced by events known as stressors that are usually identified with stimuli (or conditions) that, by definition, need to be unpredictable, uncontrollable and of forecasting uncertainty. These external or internal stimuli promote classic stress responses aimed at adaptation according to physiological and/or psychological compensation (1). Stress-associated adaptive changes may increase the resistance to pathological outcomes, thus favoring resilience, at best, or, at worst, causing dysfunctional coping that increases “allostatic load” (1, 2), leading to a disease state instead. In mammals, including humans, the brain norepinephrine (NE) and dopamine (DA) systems, originating from the locus coeruleus (LC) and the ventral tegmental area (VTA) respectively, produce spread brain networks with cortical and subcortical projections (3). Both NE and DA brainstem neurons are targeted by stress hormones of the hypothalamus-pituitary axis (HPA) (4, 5). NE-LC and DA-VTA neurons are readily activated by stressful events involving neural processes related to perception, cognitive evaluation, appraisal, and stress-dependent hormonal factors. These systems operate in parallel and in synergism, allowing to implement neural adaptations and behavioral strategies aimed at supporting resilience and overcoming stressful events (6–9). In fact, both systems are crucially involved in reward and in efforts to support motivation and coping (3, 10–12).

Brain catecholamine response to stress results in time-dependent regulatory processes involving mesocorticolimbic circuits and networks (13–16). In this context, LC-NE neurons and their projections to the cortical, thalamic, basal forebrain, and brainstem regions, including the VTA, as well as forebrain DA-targeted areas, such as the medial prefrontal cortex (mpFC) and the nucleus accumbens (NAc), appear dominant in controlling DA-dependent responses to stress (2, 17–23). In fact, stressful stimuli activate LC-NE more readily than VTA-DA neurons, which is evident by the powerful release of NE within the mPFC surpassing at large that of DA, and by the increase in tyrosine hydroxylase (TH) and Fos expression occurring within LC but not VTA neurons (13, 14, 18, 24–28).

In detail, NE in the medial pre-frontal cortex (mPFC) may produce opposite effects on DA responses, inhibiting cortical DA transmission while increasing the accumbal DA outflow being induced by first exposure to motivationally salient stimuli (17, 18). Fluctuations of accumbal DA during novel uncontrollable/unavoidable stressful experiences are tightly controlled by the opposing influences of mPFC DA and NE. Enhanced DA release in the NAc is determined by NE release on pre-frontal cortical alpha 1-adrenoceptors (α1-ARs) in a condition of low mesocortical DA activation. Instead, inhibition of NAc DA release is promoted by the return of NE to basal levels and by a sustained increase of mesocortical DA release (18, 19), as occurs in long-lasting acute or in repeated/chronic stress (2, 29, 30). mPFC NE and DA might activate two different pathways to regulate mesoaccumbens DA release in opposite ways; an ‘‘activating pathway’’ provided by indirect glutamatergic (GLUT) projections onto VTA-DA cells (31) and an ‘‘inhibitory pathway’’ provided by prefrontal GLUT efferents to VTA-GABAergic interneurons or striato-mesencephalic neurons (32–34).

Again, stress-induced LC-NE over-activation, by potentiating DA outflow in the midline thalamus, leads to rapid and persistent decrease of GABA_A_-mediated inhibitory transmission within the NAc-projecting neurons of the posterior paraventricular nucleus of the thalamus (pPVT) (23). This, in turn, promotes disinhibition of NAc-projecting neurons of the pPVT, which increases sensitivity to stress while potentiating DA efflux in the NAc shell (23, 35).

Sustained activity of LC neurons may directly potentiate the firing rate of VTA-DA neurons, mostly through α1-ARs (36–40). In addition to this direct excitation of VTA-DA neurons by LC-NE transmission acting on post-synaptic α1-ARs (36–40), an indirect stimulation is provided by inhibiting or stimulating VTA-GABA and VTA- GLUT terminals, respectively (41, 42). In fact, LC-NE may indirectly activate VTA-DA neurons by acting on pre-synaptic α1-ARs within VTA-GABA and VTA-GLUT terminals, and also on DA and GLUT terminals within the NAc (41–43). The regulation of VTA-DA neuronal activity by LC-NE inputs is quite complex. In fact, NE-induced excitation of VTA-DA neurons is followed by a long-lasting inhibition (36). This is in line with studies showing that selective lesion of LC may paradoxically increase the firing of VTA-DA neurons, though the underpinning mechanisms remain to be clarified (44). This may explain the apparent discrepancy which is present in experimental studies suggesting that stress-induced over-activity of LC-NE may exert either inhibitory or excitatory control of VTA-DA neurons (6, 45). In fact, LC-NE activity may reduce the vulnerability to emotional stress through opposite effects on VTA-DA neurons (6, 45), which may depend on the brain region and the time window of α1-AR stimulation. Such an issue remains under debate and needs experimental clarifications.

Noteworthy, stress is likely to affect catecholamine metabolism and neuroplasticity in a way which is reminiscent of the effects produced by abused substances (7, 46–48). In fact, stressful events are often reported to cause neuropsychiatric disorders going from depression to substance abuse, up to neurodegenerative insults where brain catecholamine-containing neurons and/or their projections are involved (4, 5). Chronic or heavy stress, similar to substance abuse, produces catecholamine-driven behavioral effects ranging from depression to addiction, and schizophrenia-like phenotypes. Such behavioral outcomes involving catecholamine systems are due to plastic phenomena underlying “neuronal sensitization” which in turn, is bound to alterations in the responsivity of type 1- or 2-like DA receptors (D1/D2-like DRs), and alpha/beta adrenergic receptors (α/β-ARs) (6, 16, 19, 49, 50). The occurrence of neural adaptation/maladaptation leads to specific stress-induced alterations of emotion, motivation, cognitive ability and coping.

In the latter decades, substantial attention has been paid to the role of the autophagy machinery in the physiology of catecholamine brain systems when insulted by pharmacological and neurotoxic agents (51–54). Autophagy has been recently connected with stress- and substance abuse-related disorders such as depression and addiction (51, 53, 55, 56). In keeping with this, the beneficial effects of several antidepressants and mood stabilizers are bound to autophagy activation (57–60) and autophagy inducers counteract behavioral sensitization induced by abused drugs (51, 56, 61). This wide and prolific research produced results that strongly suggest a crucial role of autophagy in response and adaptation of LC-NE and VTA-DA systems to stress. This enlightens the mechanisms by which the functional balance of the nervous system and the related behavioral and cognitive capacities are guaranteed. A few studies directly explored the connection between stress and autophagy (62–64); most information can be drawn indirectly from psychostimulants research. In the present review we discuss the role of autophagy in brain catecholamine response to stress and those factors which may lead to dysregulation.

## A Brief View of the Autophagy Machinery: From Degradation of Altered Intracellular Substrates to Modulation of Synaptic Plasticity

Autophagy is a phylogenetically conserved eukaryotic cell-clearing system that plays a primordial role in cell homeostasis (65). It is generally distinguished into macroautophagy (hereafter referred to as “autophagy”), microautophagy, and chaperone-mediated autophagy, which all promote lysosome-dependent substrate degradation (66). Beyond removing altered protein substrates, autophagy targets mitochondria, pathogens, ribosomes, portions of endoplasmic reticulum or synaptic vesicles, which are conventionally designated as “mitophagy”, “xenophagy”, “ribophagy”, “reticulophagy”, and “vesiculophagy”, respectively (66, 67). Moreover, autophagy modulates key cell functions ranging from synapse development to neurotransmitter release, and synaptic plasticity, as well as neuro-inflammation and –immunity (68, 69). A complex machinery including more than 30 autophagy-related-gene (Atg) products governs autophagy progression, starting from the biogenesis and maturation of autophagosomes up to their fusion with lysosomes. In particular, conversion of Atg8 (LC3 in mammals) into LC3-I, its ubiquitination-like enzymatic lipidation into LC3-II isoform, and eventually the incorporation of LC3-II into the phagophore membrane are critical for autophagosome assembly (59). In line with this, LC3-II is widely employed as a marker for monitoring autophagy at the morphological, ultrastructural, and biochemical level (52). However, since increased LC3-II levels may witness for either an increase or a decrease of the autophagy flux due to accumulation of stagnant vacuoles, assessment of LC3-II levels through semi-quantitative techniques can lead to results misinterpretation unless it is coupled with other autophagy markers or ultrastructural immune-labeling (52). Various additional Atg proteins ranging from Atg3 to Atg16 participate in autophagy progression *via* the processing and conjugation of Atg8/LC3 to the growing autophagosome membrane lipids (65). For instance, during Atg8 lipidation, Atg7 directly binds to and activates Atg8 fostering its transfer to the E2 enzyme Atg3. At the same time, Atg7 binds to Atg12 fostering its binding to Atg5. This leads to the formation of the Atg12-Atg5 conjugate complex, which then recruits Atg16 (65). The Atg12-Atg5/Atg16 complex localizes to the expanding phagopore where its acts as an E3 ligase mediating the final transfer of Atg8 to its lipid target phosphotidylethanolamine (PE).

The best-known autophagy-modulating pathway consists of the mTOR complex1 (mTORC1), a downstream substrate of the phosphatidylinositol-3-kinase (PI3K)/phosphatase and tensin homolog (PTEN)/AKT axis, which conveys extracellular and environmental stimuli to control cell growth, proliferation, protein synthesis and metabolism in response to bioenergetics and nutritional requests (70). Other well-known pathways that foster autophagy initiation consist of the activation of 5′ AMP-activated Protein Kinase (AMPK), and transcription factor EB (TFEB) or inhibition of glycogen synthase kinase 3 beta (GSK3-β) (58, 71).

A number of CNS disorders are characterized by dysregulated autophagy and related synaptic alterations, and/or oxidative and inflammatory processes connected with neuronal loss (51, 69). In line with this, autophagy provides neuroprotection in general, and for catecholamine neurons, which are mostly susceptible to oxidative-related alterations, in particular (49, 52, 53, 68). In fact, autophagy grants the survival of both DA- and NE-containing neurons during a variety of stressful conditions (49, 51, 52, 68, 70). Autophagy alterations are associated with the effects of abused substances (amphetamine, methamphetamine, cocaine, ethanol) on brain catecholamine neurons concerning their morphology, neuroplasticity, as well as neurotoxic and behavioral effects (51, 52, 56, 61). This is evident by a variety of behavioral effects produced by psychostimulants based on autophagy-dependent alterations (56, 61). Psychostimulants alter neuroplasticity of DA and NE neurons through receptor sensitization/desensitization while affecting their activity and metabolism (7, 49). In keeping with this, autophagy orchestrates the turnover and responsivity of various neurotransmitter receptors by intermingling with the proteasome system and intracellular trafficking and secretory pathways (66, 67). This is key to modulate neurotransmitter release while promoting either desensitization or recycling of neurotransmitter receptors to the plasma membrane. In this context it is worth noting that alterations in autophagy-dependent modulation of vesicular DA trafficking and amount of DA release contribute to maladaptive plastic changes underlying various behavioral disorders (51, 68). Conversely, autophagy induction *via* mTOR or GSK3β inhibition improves early psychomotor and cognitive alterations by rescuing neurotransmission defects in various DA-related disorders (50, 51, 59, 61, 68, 72–74).

This is not surprising since behavioral alterations are related to intracellular pathways being placed downstream of neurotransmitter receptors, which are bound to the autophagy machinery. For instance, D1/D2-like DRs, including D1DR and D5DR act as negative regulators of autophagy *via* mTOR activation (75). In detail, D1/5-DR silencing in cells lines increases LC3-II levels while attenuating mTOR activity as evident by the decrease in the levels of its downstream substrate phospho-p70-S6K, indicating activation of autophagy (75). Opposite results are obtained following D1/5-DR overexpression (75). Remarkably, when D1/5-DR silencing is combined with administration of the autophagy flux blockers bafilomycin/chloroquine, the latter are less effective in inhibiting autophagy compared with negative controls, as shown by higher LC3-II and lower phospho-p70-S6K levels (75). This suggests that D1/D5-DRs may exert a powerful negative control on the autophagy machinery. Opposite results were obtained for D2-likeDRs, indicating that they act as autophagy stimulators through AMPK activation and mTOR inhibition (75). This was confirmed in several cells lines, including TH-positive primary midbrain neurons, where DRD2 and DRD3 activation by pramipexole and quinpirole promotes beclin 1-depedent autophagy activation (76). This is associated with neuroprotection and inhibition of alpha-synuclein/SNCA accumulation both in rotenone-treated catecholamine-containing cells that overexpress wild-type or mutant alpha-synuclein and in *SNCA* transgenic mice (76). More recently, D3DRs were shown to be specifically responsible for autophagy activation *via* AMPK stimulation and mTOR inhibition (77).

Despite such an evidence suggesting that D1-likeDRs may block while D2-likeDRs may promote the autophagy flux, further *in vivo* confirmatory studies are needed.

Autophagy is also variously altered by the signaling pathways cyclic AMP (cAMP)/protein kinase A (PKA)/protein kinase C (PKC) and TFEB/peroxisome proliferator-activated receptor gamma coactivator 1-alpha **(**PCG-α) which are triggered downstream of ARs (78–83). In particular, β2-ARs may induce autophagy, which is associated with NE-related protection (79, 82, 83). For instance, agonist-induced β_2_‐ARs activation prevents disruption of autophagy flux in skeletal muscle of mice with neurogenic myopathy, which is associated with improved skeletal muscle proteostasis and contractility properties (83). Autophagy blockade through either chloroquine or skeletal muscle‐specific deletion of *Atg7* abolishes the beneficial effects of β_2_‐ARs activation. Similarly, administration of the β_2_-agonist clenbuterol stimulates the autophagy flux in hepatic cells, while the β_2_-antagonist propranolol produces opposite effects (79). These effects were confirmed by co-administering chloroquine and through both biochemical (LC3II and p62 quantification) and ultrastructural analyses (79). Nonetheless, the potentially beneficial effects of NE-induced autophagy remain to be confirmed.

## Stress and Bidirectional LC-VTA Communication: Potential Role of Autophagy

Abused psychoactive substances and stress engage shared DA and NE neural mechanisms, as shown for instance by numerous studies pointing to a reciprocal cross-sensitization (84, 85). Stress, similar to abused substances, strongly stimulates LC-NE and VTA-DA transmission, producing an activity overload that brings into play adaptation mechanisms based on feedback circuits between connected neural systems and molecular adjustments in the cells. When stress persists, these compensatory mechanisms fail to restore an ante-stress balance and foster neurodegeneration (6, 82, 86). In fact, during chronic/prolonged stress or drug abuse, a dysregulation of LC-VTA connectivity may occur, decreasing catecholamine release due to diminished LC and VTA activities (9, 86–92). While early being associated with apathy and depression, stress- or drug-induced alterations within LC and VTA may predispose to cognitive decline and neurodegeneration (86, 93). Here we consider available evidence to cast the hypotheses that (i) stress or drug-induced LC-NE overload may alter VTA-DA neurons activity, plasticity and metabolism; (ii) DA overload may in turn lead to a progressive reduction of LC activity that occurs during chronic stress; (iii) a reduction of LC activity may occlude the neurotrophic and neuroprotective effects of NE in LC projecting areas, including the VTA. These functional links between DA and NE brain systems suggest a crucial role for the catecholamine network in adaptive behavior and in stress demands to which the organism has to cope with. At the same time, some contradictory findings may be explained by the double-faceted effects of NE in the brain. While at physiological levels NE exerts neuro-protection, abnormally increased NE levels may induce apoptosis and neurodegeneration (94). In this frame, the autophagy machinery, as a major pathway that regulates both neuronal proteostasis and synaptic plasticity, may be involved in various steps of catecholamine systems’ response to stress and drug intake/administration.

### LC-NE and VTA-DA Transmission Overload

Brain DA and NE systems are connected through cortical-subcortical circuitry as well as through direct bidirectional LC–VTA pathways. The two-way communication between LC and VTA is key for drug-induced reward and reinforcement underlying maladaptive synaptic plasticity in striatal, limbic and cortical brain areas (7, 95). In detail, NE from the LC potently regulates drug-induced reward and reinforcement by stimulating DA release mostly within the ventral striatum (96). This may occur either directly or indirectly since NE-containing axons of the LC project to both VTA, and likely, the NAc shell (97). While DRD1-expressing medium spiny neurons of the NAc medial shell directly inhibit mesolimbic VTA-DA neurons, NAc lateral shell neurons mainly project to the VTA-GABA neurons to disinhibit VTA-DA neurons (98–100). These in turn, fire back to both the NAc lateral shell (98) and the LC (101). Selective stimulation of NAc lateral terminals in the VTA induces a potent reward phenotype, which is likely caused by a disinhibition of VTA-DA neurons (98). Acute exposure to stress, similar to drugs of abuse, alters inhibitory plasticity which may increase VTA excitability (42, 84, 100, 102). This occurs by blocking the induction of long-term potentiation at GABA_A_ synapses while increasing GLUT release on VTA neurons (42, 43, 84, 100, 102).

In this scenario, autophagy is a key by acting at the level of both GABA and DA systems (103–106). Conditional deletion of *Atg7* in GABA inhibitory or excitatory neurons, similar to what observed in Unc-51 Like Autophagy Activating Kinase 2 heterozygous (*Ulk2*+/-) mice, leads to autistic-like behavioral abnormalities including social deficits, increased distress and anxiety along with cognitive alterations (103, 104). In detail, autophagy deficiency within GABA neurons brings to hyper-excitability due to reduced membrane expression of GABA_A_ receptors. In fact, these receptors are entrapped within SQSTM1/p62-positive aggregates (103, 104). Autophagy activation replaces GABA_A_ receptors on the plasma membrane thus reducing abnormal hyperexcitability (104). At behavioral level this is evident by rescuing behavioral deficits in *Ulk2*+/- mice (104). Again, mice lacking *Atg7* specifically within DA neurons display increased evoked striatal DA secretion along with decreased DA re-uptake (105, 106). In line with this, activation of mTOR-dependent autophagy decreases evoked DA release in wild-type but not in transgenic mice (105). Thus, an impairment of mTOR-dependent autophagy at the synapse fosters unrestrained DA release (105). Consistently with this, abused substances activate mTOR signaling in the mesolimbic reward circuit while administration of the mTORC1 inhibitor and autophagy activator rapamycin reverses drug-induced relapse and reinforcement (61, 107–110). For instance, systemic treatment with rapamycin, similar to the infusion of lentivirus-expressing mTOR-shRNA into the NAc shell, suppresses the induction of methamphetamine-induced sensitization while rescuing morphological alterations in the NAc’s dendritic spines (61). Again, selective deletion of *mTOR* within mouse VTA counteracts drug addiction by decreasing DA release in the NAc through potentiation of VTA-GABAergic neuron firing (110). Thus, mTOR-dependent autophagy regulates drug action by modulating both DA and GABA signaling within the VTA and subsequent DA release within target brain areas. This indicates that an autophagy impairment within the VTA may strengthen the feedback loop in which the VTA fires to the NAc, and back to the LC. In this way, the LC would then feedback into the VTA *via* α1-ARs to further evoke DA release in the NAc, potentially sustaining behavioral sensitization (7, 39).

### Sustained LC-NE Transmission Predisposing to Oxidative-Related Neuronal Alterations Within VTA

When dealing with the multiple effects of α1-ARs, we may summarize that LC-NE transmission increases VTA-DA neurons activity both directly and indirectly by acting on α1-ARs within i) VTA-DA neurons, ii) GABA and GLUT terminals within the VTA, iii) DA and GLUT terminals in the NAc and mPFC (36–63). A crucial role of α1-ARs stimulation in the VTA by LC-NE is documented for the neurochemical and reward processes of abused substances, which leads to an increase in DA release through LC projections to VTA and NAc shell (7, 39, 47, 97). Overlapping with the effects of acute stress and drug exposure, activation of presynaptic α1-AR within the VTA depresses GABA while enhancing GLUT release and increasing AMPAR/NMDAR ratios within VTA-DA neurons (41, 42, 84, 111). Although different brain nuclei being targeted by LC-NE are known to serve as a source of GLUT to the VTA (including the prefrontal cortex and the bed nucleus of stria terminalis), α1-AR -induced GLUT inputs into the VTA seems to derive mostly from local GLUT neurons (41, 112, 113). While contributing to stress- and/or drug-induced behavioral alterations (41, 84, 111–113), an excess of α1-ARs-induced GLUT release onto VTA may increase its vulnerability to Ca2+-related excitotoxicity. In fact, stress- and drug-induced GLUT release onto VTA neurons is coupled to the calcium (Ca2+)-related PKC signaling pathway (41), which produces amphetamine-related oxidative damage going along with autophagy impairment (114, 115). In this context, autophagy is implicated in both GLUT-dependent synaptic plasticity and excitotoxicity. Transient exposure to low doses of NMDA induces autophagy through PI3K/AKT/mTOR pathway inhibition, which is key to promote AMPAR degradation in cultured rat hippocampal neurons and in rodent models of auditory fear reconsolidation (116, 117). On the other hand, neuroprotection against GLUT excitotoxicity is achieved by administering either mTOR-dependent or -independent autophagy inducers rapamycin or trehalose (118). This is in line with evidence showing that NMDAR antagonists may rescue autophagy flux and mitophagy to confer neuroprotection (119). These findings suggest that an autophagy failure being bound to either impaired degradation of plasma membrane AMPA receptors or NMDAR-mediated Ca2+ signaling, may be implicated in the responsivity of VTA neurons to α1-AR-induced GLUT release.

Again, the coupling of α1-AR signaling and the stress hormone corticotrophin-releasing factor (CRF) produces social stress enhancement of drug conditioning *via* NMDAR-mediated GLUT transmission within the VTA (8). Intriguingly, this is coupled to an amplification of IP_3_-Ca^2+^signaling, which is known to impinge on the autophagy pathway (57, 59). Within LC neurons, constitutive overexpression of CRF increases NE activity and redistributes beta-amyloid (Aβ) peptides from synapses to somato-dendritic processes, which occurs along with altered distribution and morphology of autophagy-related vacuoles (120). Again, CRF was recently shown to inhibit the autophagy pathway *in vitro* (121), suggesting that α1-AR and CRF stimulation following abnormal NE release may synergize to alter autophagy within VTA.

These findings are also in line with evidence on a deleterious role of high NE levels, which similar to stress/drug exposure, do occur in REM sleep deprivation (94, 122–124). In *ex vivo* and *in vivo* models of REM sleep deprivation, high NE levels lead to iron and calcium-related oxidative damage within neurons and glia *via* α1-ARs, which is accompanied by mitochondrial failure and altered levels of AKT (94, 122–124). This occurs in various brain regions including the LC itself, though the VTA remains to be examined. In this frame, it is likely that stress- and drug-induced catecholamine alterations may increase the susceptibility of LC and VTA to neuronal damage by increasing the formation of highly oxidative DA- and NE-derived metabolites, which are known to impair neuronal proteostasis (125, 126). This would explain why catecholamine-containing neurons are particularly susceptible to degeneration associated with an autophagy failure (127–129).

**Figure 1:**
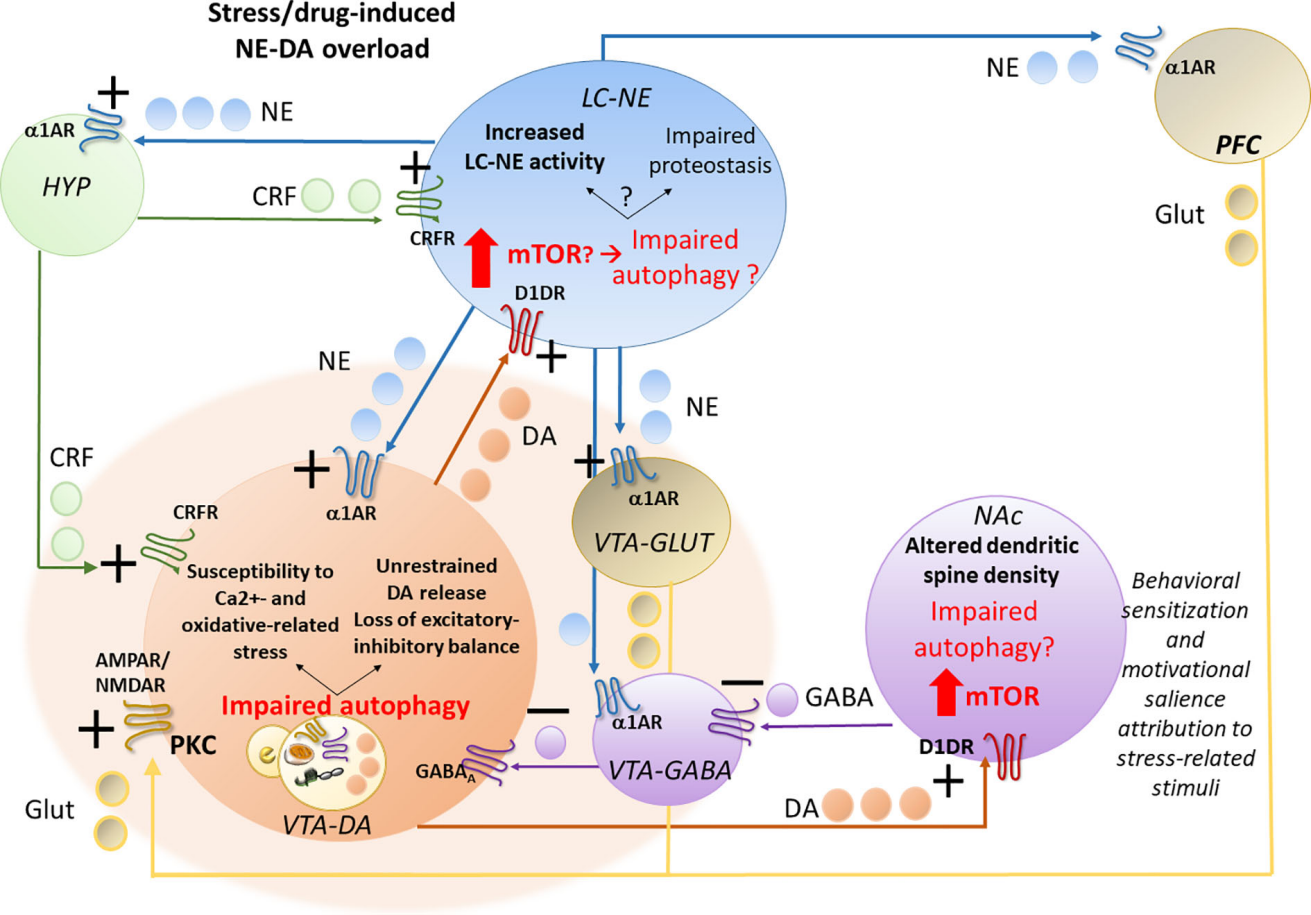
Acute stress- or drug-induced potentiation of locus coeruleus–norepinephrine (LC-NE) and ventral tegmental area–dopamine (VTA-DA) activity and potential role of autophagy. Stress, similar to abused drugs, readily activates LC-NE and VTA-DA neurons, leading to a sustained NE transmission and increased NE release in the hypothalamic CRF-producing neurons, in the forebrain DA-targeted areas pre-frontal cortex (PFC) and nucleus accumbens (NAc) and also directly on VTA neurons. In fact, LC-NE excites VTA-DA neurons either directly through post-synaptic α1-ARs, or indirectly, through inhibition of VTA-GABA neurons and activation of VTA-GLUT neurons *via* stimulation of pre-synaptic α1-ARs. Again, NE in the PFC, through α1-ARs stimulation, might activate DA release on the NAc through GLUT cortical projections to VTA-DA cells. While altering the excitatory-inhibitory balance and increasing the metabolic rates within VTA-DA neurons, these NE-mediated events may predispose to oxidative stress, and glutamate-related alterations, which may in turn contribute to overwhelming autophagy. While increasing VTA neurons susceptibility to oxidative and Ca2+-related alterations, autophagy impairment within VTA neurons promotes an empowering of DA release to the NAc and likely, also back to the LC. In this way, through stimulation of post-synaptic D1DRs, which is known to promote mTOR activation, DA overload may in turn impair autophagy within the NAc, where it alters dendritic spine density while contributing to drug-induced behavioral sensitization and likely, altered motivational salience attribution to stress-related stimuli. VTA-DA overload, through stimulation of post-synaptic D1DRs, may also impair autophagy within the LC. This may lead to either potentiation of NE release or a progressive impairment of proteostasis impinging on LC neuronal integrity, which remains to be investigated.

These findings support a correlation between early potentiation of NE-DA activity and autophagy-related alterations within the LC-VTA network following stressful stimuli or exposure to addictive substances (**Figure 1**).

## Chronic Stress and Drug Exposure Bridging Reduction of NE-LC and VTA-DA Activity and Autophagy-Dependent Neuroprotection

### Reduction of NE-LC Activity May Occur Due to DA Overload

Dysregulations of NE activity during exposure to prolonged/chronic stress or abused drugs may include either an increase or a decrease of LC activity. In fact, cumulative cocaine self-administration in rats leads to functional reductions in the LC (92), and a decrease in baseline LC neuron activity and NE release occurs in rodent models of post-traumatic stress disorder - single prolonged stress (SPS), chronic unpredictable mild stress (CUMS), as well as chronic social defeat stress (CSDS) (9, 86, 92). The activation state of VTA-projecting LC-NE neurons, and the amount of NE released into the VTA are critical for determining the vulnerability to emotional stress (45). The loss of NE neurons projecting to the VTA leads to a potentiation of VTA-DA firing conferring an increased susceptibility to stress induced by social defeat compared with resilient mice (45). This is in line with evidence showing that selective lesion of LC increases the firing activity VTA-DA neurons (44). Since VTA-DA excites LC-NE neurons (101), it is expected that exaggerated firing of VTA neurons occurring during acute/repeated stress or abused substance intake/administration, may progressively impinge on LC thus altering this nucleus. In this context it would be worth investigating whether LC alterations are bound to VTA-DA-induced stimulation of D1DRs within the LC, which is essential for ethanol-triggered reinforcement behavior (48). This would be key in the light of evidence indicating that D1DRs that occur within LC neurons (48), may impair autophagy (75). Thus, similar to NE-dependent alterations affecting the VTA discussed in 3.2, autophagy may be implicated in DA-dependent alterations within the LC. In this scenario, a protective role for NE, which may act as an autophagy inducer (79, 82, 83), is substantiated by evidence showing that LC-NE dysfunction may predispose to degenerative phenomena involving various LC-NE-targeted brain areas (82, 86). Here we hypothesize that these may include DA-containing neurons, where reduction of LC-NE release could contribute to undermining intracellular protection mechanisms.

### Impairment of LC-NE to VTA-DA Neurons May Occlude NE-Induced Protective Autophagy

Neuroprotective effects of the LC-NE on DA neurons *in vivo* and *in vitro* have been documented emphasizing the effects of NE as a neurotrophic factor and its ability to stimulate the expression of other neurotrophic factors (28, 130–134). This is the case of brain-derived neurotrophic factor (BDNF), which is synthesized within glial cells or neurons mainly through β1/β2-ARs (133, 134). In fact, β2AR agonists reverse DA neurotoxicity *in vitro* and *in vivo* (130, 133). As shown in mice models, this occurs through the inhibition of microglial activation rather than exerting a direct effect on VTA-DA neurons, which in fact lack β2-ARs (133). NE-induced neuroprotection of DA neurons depends on the presence of β2AR complexed to β-arrestin (133), which intriguingly, mediates neuroprotection in experimental cerebral ischemia through coordination of BECN1-dependent autophagy (135).

In line with this, there is evidence indicating that NE induces protective autophagy mostly through β2-AR stimulation (79, 82, 83), and again, the protective effects of BDNF are bound to autophagy activation. In detail, BDNF works through inhibition of either mTOR (136) or GSK3β pathway (137), which are main upstream regulators of autophagy. *In vivo*, BDNF enhances autophagy flux and promotes mitophagy through the HIF-1α/BNIP3 pathway (138). BDNF and related autophagy ameliorate stress-induced behavioral and emotional alterations, suggesting that a direct association exists between autophagy impairment and BDNF deficiency (136, 138). This is not surprising since autophagy has been implicated in the regulation of neurogenesis, which is altered by psychological stress and represents a risk factor for the development of mood/neuropsychiatric disorders (63). In line with this, the administration of either antidepressant drugs or the naturally occurring autophagy inducer resveratrol alleviates depressive-like behavior in mice models of CUMS or post-partum depression by increasing BDNF and autophagy-associated proteins (136, 138). This goes along with reduced HPA axis hyperactivity, CRF and pro-inflammatory cytokines levels (136, 138). In line with this, blockade of autophagy by chloroquine abrogates whereas the autophagy inducer rapamycin protects against the pro-inflammatory effects of CRH in other mice tissues besides the brain (139). These findings suggest that VTA-DA neurons may benefit from glial β2-AR stimulation and BDNF/autophagy induction through reduction of CRF and microglial-mediated inflammation. Autophagy failure is expected to occlude the neurotrophic and anti-inflammatory effects of NE. In fact, microglia-specific *Atg5*-deficient mice show higher inflammation levels, reduced BDNF expression, and exacerbated depressive-like behavior compared with wild-type mice (138). However, the link between autophagy, NE levels and stress/drug-related behavior remains to be investigated.

### VTA-DA Activity Decline in Chronic Stress and Drug Abuse: Potential Role of Autophagy and Future Issues to Address

As discussed above, an impairment of autophagy within VTA-DA neurons is expected to occlude neurotrophic and neuroprotective mechanisms depending in part on LC-NE activity. Remarkably, an early loss of VTA-DA neurons, which precedes LC neuronal degeneration, has been documented in models of Alzheimer’s disease (93). The potential mechanisms underlying such a temporal dissociation between LC and VTA degeneration will not be dealt herewith. Here, we wish to point out that different patterns of stress exposure might induce hypo-DAergic states (88–90). In detail, acute stress readily activates whereas chronic stress exposure may lead to a compensatory downregulation of the DA system (88–90). In this context, also the timing of stress is critical since contrarily to adolescent stress, adult stress induces a depression-like hypo-DAergic state (140). Similarly, chronic drug intake/administration is associated with a decrease in DA release mostly within the striatum, which may explain the decreased sensitivity to natural rewards and the compulsive drug use as a means to temporarily compensate for this deficit (87). Such an effect is associated with reduced striatal levels of D2-/D3-DRs (87, 141, 142). In the light of an interdependency between autophagy, substance abuse, and D2-/D3-Rs activity discussed in section 3, a potential link deserves to be investigated in stress-related disorders. A reduction of DA activity may be the outcome of either abnormally increased LC-NE release predisposing VTA-DA cells to excitotoxicity, or LC neuronal loss occluding the protective effects of NE upon VTA-DA neurons, as discussed in sections 3.2 and 4.2, respectively. This may be explained by the double-faceted effects of NE in the brain, exerting neuro-protection at physiological levels, while inducing apoptosis at high concentrations (94, 122–124).

Chronic exposure to psychosocial stressors in adults seems to be associated with reduced striatal DA synthesis, mostly within the ventral striatum (90). Autophagy and stress-related VTA-DA alterations may be reminiscent of the molecular effects produced by psychostimulants, which alter autophagy vacuoles (50, 143). This hypothesis remains to be confirmed and may represent the starting point to explore the effect of stress at subcellular level within VTA-DA cells. Despite appearing in contrast with evidence indicating that autophagy blunts DA transmission, these findings suggest that autophagy regulation may be a finely-tuned and context-dependent process, depending on specific patterns of neuronal activity and metabolic demands. In fact, an autophagy impairment within different cell compartments may have different effects, for instance, driving neuropathological changes at the soma while producing striatal-driven behavioral changes by increasing the extracellular availability of DA at the synapse (106).

## Conclusions

The evidence here discussed points to the remarkable action of autophagy in NE-LC and VTA-DA connections and its role in NE-dependent neuroprotection, which is crucial for the organism adaptive response to stress and allostatic load. In fact, heavy and/or chronic stress is known to induce or foster neurodegeneration in brain catecholamine neurons involved in a number of psychiatric and neurodegenerative diseases. We proposed here the autophagy machinery as a relevant mechanism of regulation and dysregulation of catecholamine neurons. As for NE-containing neurons, autophagy is seminal for the survival of DA neurons and remarkably, it plays a central role in DA release. The role of autophagy in brainstem NE and DA neurons and their projections in response to psychostimulants indicates adaptive mechanisms that can be activated by stress following its impact on neurotransmission. LC and VTA are both crucial in the response of the organism to stressors and both orchestrate brain systems involved in the appraisal and in the management of psychological stress. Lessons from the effects of abused substances suggest that autophagy, with its role in catecholamine synapse and in neuronal protection, is crucial in modulating the effects of psychological stress on emotional and cognitive driven behavior to foster adaptive emotional outcomes aimed to wellbeing.

At cellular level, psychological stress may translate into intracellular stress-responsive events, such as ER- and oxidative- stress and inflammation (144), which are known to promptly recruit autophagy in the attempt to restore homeostasis. However, under conditions of chronic/persistent stress, when alterations in neurotransmitter activity translate into maladaptive neuronal changes, autophagy may be consistently affected, fostering progressive synaptic deterioration up to neurodegeneration. In the light of an interdependency between autophagy and the mechanisms governing neurotransmission and neuronal homeostasis, we strongly believe that the role of autophagy deserves to be further investigated in the context of catecholamine response to psychological stress specifically.

The possibility of relating stress-related emotional and cognitive experiences to functional aspects through molecular pathways, may facilitate the discovery of potential biomarkers identifying an early risk. Individual differences in the response to stress and autophagy activity, which can make some individuals more or less susceptible than others, should also be addressed by future studies.

## Author Contributions

SP-A, FL, and FF drafted and wrote the manuscript. FL, CB, and FB contributed to literature review, manuscript editing, and art-work. SP-A and FF are coordinators of the paper; they critically revised the article for important intellectual content.

## Funding

The present research was funded by Ministero della Salute (Ricerca Corrente 2020).

## Conflict of Interest

The authors declare that the research was conducted in the absence of any commercial or financial relationships that could be construed as a potential conflict of interest.
